# Supplementary material for: Optimization of subsampling, decontamination, and DNA extraction of difficult peat and silt permafrost samples
Source: Sci Rep. 2020 Aug 31;10:14295. doi: 10.1038/s41598-020-71234-0 (PMC7459103; doi:10.1038/s41598-020-71234-0)
Supplement: Supplementary file 1 — Supplementary file1 [file 41598_2020_71234_MOESM1_ESM.pdf]

SUPPLEMENTARY INFORMATION

Optimization of subsampling, decontamination, and DNA extraction of difficult peat and silt permafrost samples

Alireza Saidi-Mehrabad[1], Patrick Neuberger[1], Maria Cavaco[1], Duane Froese[2], and Brian Lanoil[1,*]

[1] Department of Biological Sciences, University of Alberta, Edmonton, Alberta, T6G 2E9, Canada

[2] Department of Earth and Atmospheric Sciences, Edmonton, Alberta, T6G 2E3, Canada

*Correspondence: brian.lanoil@ualberta.ca

SUPPLEMENTARY INFORMATION

TABLE S1. Previously published permafrost decontamination, DNA extraction and sample handling protocols

Decontamination protocol	DNA extraction protocol	Spike	Sample handling[g]	Year	Reference
Scraping	CTAB	BE[b]	C, E	2006	(9)
	N/A[a]	-[c]	N/A	2006	(10)
	Marmur	AE[d]	N/A	2007	(11)
	Chloroform: octanol	BE	A, C, D	2007	(12)
	PowerSoil	-	N/A	2010	(13)
	CTAB	-	N/A	2011	(14)
	PowerSoil	-	N/A	2013	(15)
	PowerSoil	-	E	2014	(16)
	CTAB/phenol: chloroform	AC[e]	N/A	2015	(17)
	PowerSoil	BE	B	2016	(18)
	PowerSoil	-	E	2018	(19)
Disk sampling	Chloroform: isoamyl alcohol	BE	N/A	2000	(20)
	PowerMax	AE	N/A	2010	(21)
	TCEP	BC[f]	A, C	2011	(4)
	N/A	BE	N/A	2012	(22)

	N/A	-	N/A	2013	(3)
	Phenol: chloroform	-	N/A	2014	(23)
Scraping and disk sampling	Chloroform: octanol	BE	A, C, D	2005	(24)
	PowerSoil	-	N/A	2011	(25)
	PowerSoil	BE	N/A	2015	(26)
	PowerSoil	-	N/A	2015	(27)
	Fast DNA	AC	N/A	2017	(28)
Not determined	Marmur	-	N/A	2005	(29)
	Fast DNA	-	N/A	2007	(30)
	PowerSoil	-	N/A	2009	(31)
	PowerMax	-	A	2013	(32)
	Freeze grinding mechanical lysis	-	N/A	2013	(33)
	Chloroform fumigation and K_2SO	-	C	2014	(34)
	PowerMax	-	N/A	2014	(35)
	PowerMax	-	N/A	2015	(36)
	Freeze drying and chemical lysis	-	C	2015	(37)
	PowerSoil	-	C	2018	(8)
	Fast DNA	-	E	2018	(38)
	PowerSoil	-	N/A	2018	(39)

[a]N/A = not mentioned.
[b]BE = A biological spike applied to coring equipment.
[c]- = Not used.
[d]AE = An artificial spike applied to coring equipment.
[e]AC = An artificial spike directly applied to the permafrost cores.
[f]BC = A biological spike directly applied to the permafrost cores.
[g] A = A clean lab was used, B = Sterile tools were used, C = Clean reagents with controls were used, D = The experiment was replicated in another university, E = Other (Use of a mobile hood, anaerobic glove box or a soil press).

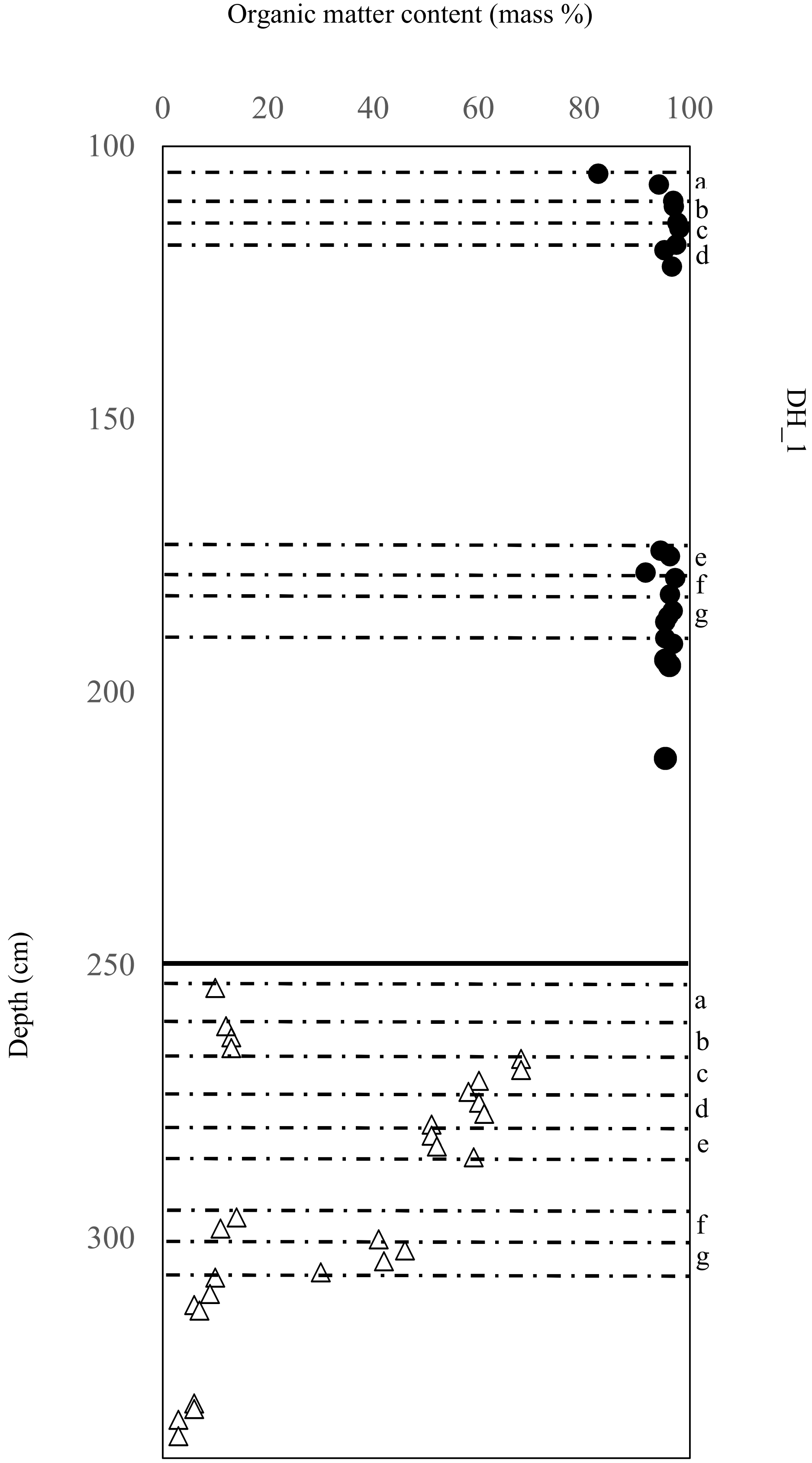

Organic matter content (mass %)
0
20
40
60
80
100
Depth (cm)
100
150
200
250
300
a
b
c
d
e
f
g
DH_1
a
b
c
d
e
f
g

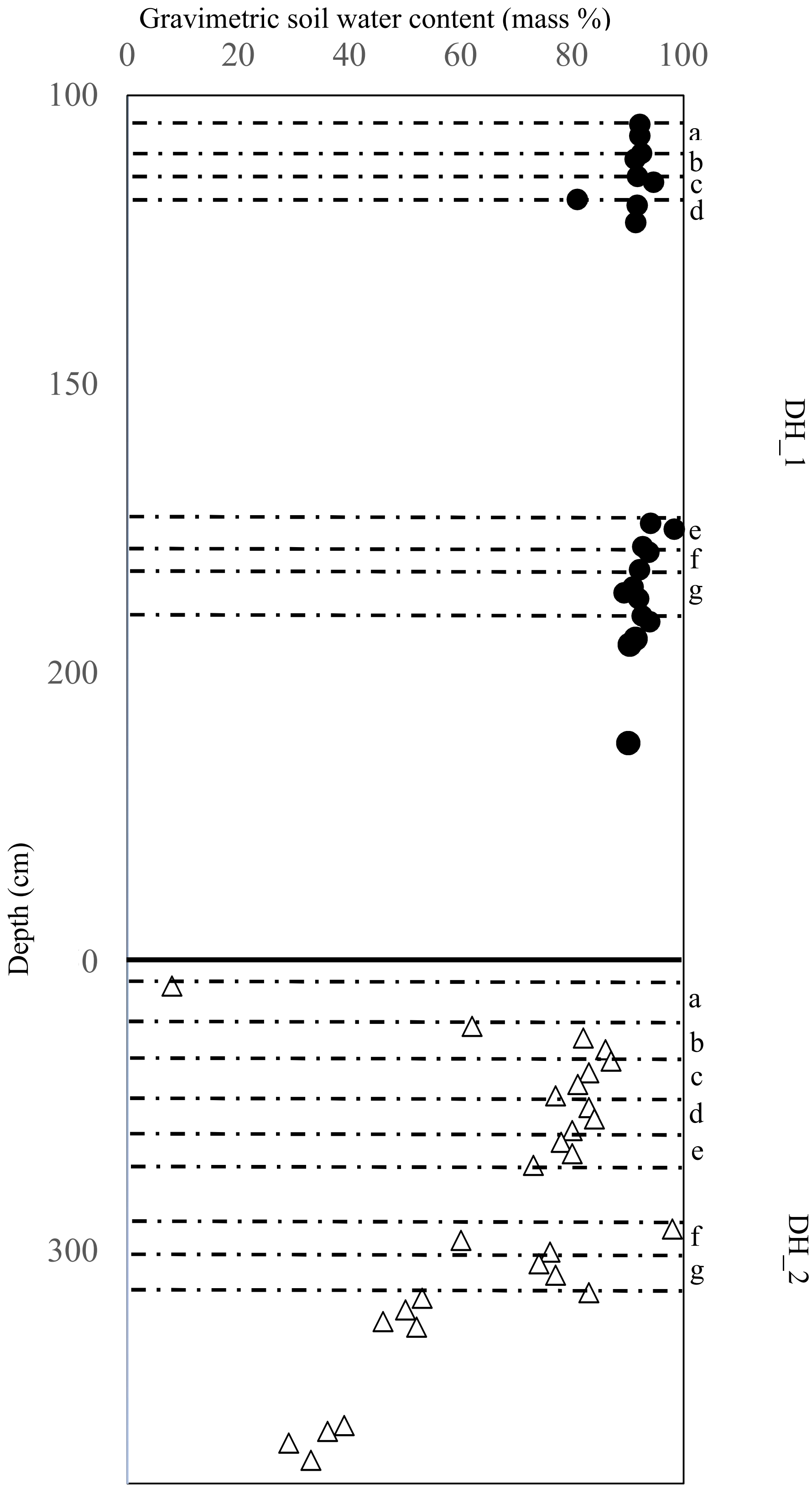

Gravimetric soil water content (mass %)
0
20
40
60
80
100
Depth (cm)
100
150
200
0
300
a
b
c
d
e
f
g
DH_1
a
b
c
d
e
f
g
DH_2

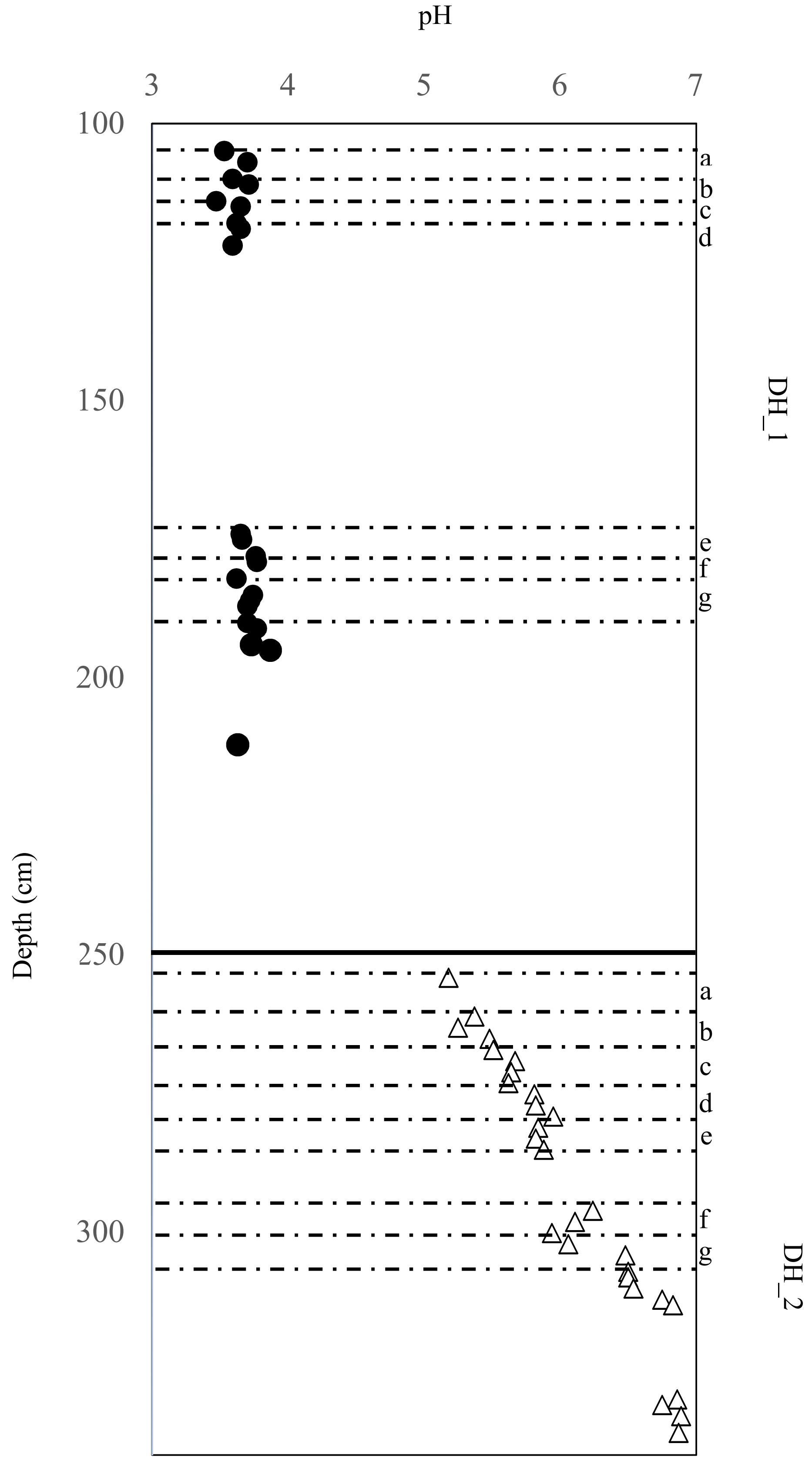
pH
3
4
5
6
7
100
150
200
250
300
Depth (cm)
a
b
c
d
e
f
g
DH_1
a
b
c
d
e
f
g
DH_2

FIGURE S1**.** Chemical profiles of DH_1 and DH_2 permafrost samples in relation to depth. Depth (cm) is relative to the permafrost table. Dashed lines indicate depth of sample used for noted decontamination protocol labeled as in Table 2. The solid line depicts the boundary between permafrost with high peat (DH_1, filled dots) and high silt content (DH_2, hollow triangles). A. Organic carbon content (mass %), B. Gravimetric water content (mass %), C. pH. The observed gap between sample d and sample e for DH_1 is due to the presence of ice pockets or not measured.

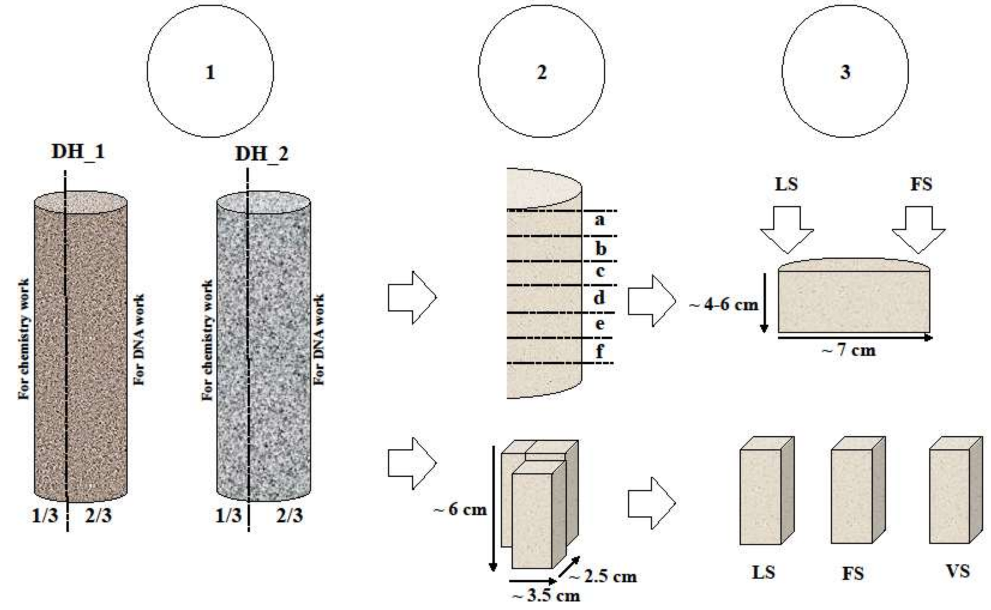

FIGURE S2. Sample preparation for decontamination protocols (a-g). Step 1: Initial dry cut. DH-1 and DH-2 cores cut into 1/3 and 2/3 sections. Step 2: Cutting into subsections. The upper portion of the ⅔ section cut horizontally for decontamination protocols (a-f). For protocol (g), the ⅔ section was cut into three rectangular pieces. Step 3: Intentional contamination of the samples. For protocols (a-f), one side of the sample was painted with the spike in the lab (LS) and the other side only contained the sprayed field spike (FS). For protocol g, one rectangular piece only contained the sprayed field spike (FS); one piece was painted with the spike in the lab (LS), and one piece was painted with pure vector (VS).

SUPPLEMENTARY METHODS

Sterilization procedure of the tools and the work stations

All decontamination methods were performed in a class 1000 clean laboratory separate from the DNA extraction and PCR labs, and with no prior history of DNA work (1, 2). The surface of the working area was washed with 100% concentrated bleach (Clorox, California USA) followed by 70% ethanol (1). Later the clean area was covered with bleach (Clorox, California USA) and 70% ethanol treated heavy duty aluminum foil (AlCAN Plus, Canada). During decontamination protocol testing, full Tyvek body suits with elasticized hood (Bennett, ON Canada), standard polypropylene shoe covers (WorkHorse, Ohio USA), N95 standard face masks (3M, Minnesota USA), powder free Sterling nitrile gloves (Kimberly-Clark, Texas USA), and standard safety goggles were worn. 70% ethanol treated gloves were changed regularly during the decontamination process. All the metal objects and glassware were muffle furnace baked at 450°C for 5 h. Chisels were burnt with 70% ethanol with a hand torch prior to contact with the samples. Milli Q water used for washing core segments was pre-filtered through Millex-GS 0.22 μm syringe filters (Millipore Canada Ltd, Ontario Canada) and autoclaved twice for 30 min prior to use. Metal rulers and hand saw were cleaned with 70% ethanol and the working area in the walk in 4°C cold room was surface cleaned with 100% concentrated bleach (Clorox, California USA) followed by 70% ethanol. Half strength and full strength Luria Bertani (LB, BD Difco manual) and nutrient agar (NA, Thermo Fisher Scientific, Canada) plates without lids were employed to test for the presence of the aerosolized bacteria before, during and after the decontamination procedures (modified from (3, 4)).

All DNA extractions were performed in an EdgeGARD Horizontal Laminar-Flow Clean Bench (model EG6220, Baker, Maine, USA) decontaminated with 100% concentrated bleach (Clorox, California USA) followed by 70% ethanol. All forceps and spatula were soaked in 70% ethanol and flame sterilized for ~5 s. All the plastic racks for holding the tubes were washed and kept in 10% bleach (Clorox, California USA) for 24 h prior to DNA extraction. Certified RNase/DNase and pyrogen safe Filter pipet tips (Axygen Scientific, California USA) were used in every step of the extraction procedure and all the hand pipettes were cleaned with 70% ethanol prior to any experiment.

Basic chemical parameter analyses of the core segments

Gravimetric water content was measured by oven drying at 100°C for 48 h (5), organic matter content was determined based on loss on ignition at 450°C for 5 h in a standard muffle furnace (6). pH was measured with the aid of an AB15 pH meter (Fisher Scientific, Canada) by buffering the samples with 0.01 M $CaCl_2.2H_2O$ at a 1:2 soil: water ratio (7).

Modified DNA extraction protocols

Protocol 2: FastDNA SPIN Kit for Soil

Samples were homogenized for 45s at 2000 ×g twice. After adding the Binding Matrix suspension, the samples were placed on a rotor to allow DNA to bind for 15 min and later centrifuged for 1 min at 13,000 × g to remove the silica matrix. The washing solution in

the spin filter was evaporated by incubating the tubes in a heat block at 70°C for 15 min. 50 µl of water was used instead of 100 µl for the final elution step.

After confirming that the DNA concentrations were below the detection limit of the Qubit, eluted DNA samples were pooled and mixed for a second time with 4 ml Binding Matrix. The conical tubes were inverted by hand for 2 min to allow binding of DNA and later placed in a rack for 20 min to allow settling of the silica matrix. 600 µl of the mixture was added to new set of spin filters and centrifuged at 13,000 ×g for 20 min, which was repeated until all samples passed through into the catch tubes. Then we followed the remaining steps as described by the manufacturer's protocol. The final eluted DNA samples were subjected to Zymo Genomic DNA Clean & Concentrator-10 (Zymo Research, California, USA) kit by following the manufacturer's instructions.

Protocol 5: PowerSoil DNA Isolation Kit

At step 14, solution C4 was added in a total of 4 ml instead of 1200 µl and the vortexing step was 20 seconds. The samples were incubated for 30 min prior to step 15. At step 20 instead of 100 µl, the DNA was eluted in 50 µl of solution C6.

Protocol 7: ZymoBIOMICS DNA Microprep Kit

During the DNA extraction, we did not use the Zymo-Spin IV Spin Filters. All the supernatants after the bead beating and centrifugation steps were pooled into a single 50 ml conical tube. For optimal performance, 0.5% beta-mercaptoethanol (v/v) was added to the ZymoBIOMICS DNA Binding Buffer prior to use. Three times the volume of ZymoBIOMICS DNA Binding Buffer was used to maintain the ratios. The samples were

incubated for 30 min at room temperature with vigorous shaking. The entire DNA binding buffer was passed through 3-4 Zymo-Spin IC-Z columns. After each washing step, samples were centrifuged for extra 3 min to remove the excess ethanol. After adding 10 µl of ZymoBIOMICS DNase/RNase free water the samples were allowed to incubate for 5 min. The final centrifugation step was for 5 min.

Contamination detection

The presence of the tracer was tested with pBAD-forward (5′ATGCCATAGCATTTTTATCC3′) and pBAD-reverse (5′GATTTAATCTGTATCAGG3′) primers (Invitrogen, Canada). The 50 µl PCR reaction included: 1× PCR buffer (Invitrogen/ThermoFisher, Canada); 200 µM dNTP mixture (Invitrogen/ThermoFisher, Canada); 0.5 µM each primer; 1.5 mM $MgCl_2$ (Invitrogen/ThermoFisher, Canada); 1.25 U Platinum Taq DNA polymerase (Invitrogen/ThermoFisher, Canada); 36.25 µl of H_2O (Integrated DNA Technologies, Iowa, USA); and 1-2 µl of 1:2, 1:10 or 1:100 diluted DNA template. The program for amplification included: 95ºC for 50 s, followed by 33 cycles of 94ºC for 20 s, 44ºC for 30 s, and 72ºC for 1 min, with a holding temperature of 4ºC.

16S rRNA gene-targeted PCR protocol

The 16S rRNA gene PCR reactions were performed in a Veriti 96 well thermal cycler (Applied Biosystems, Thermo Fisher Scientific, Canada) using primers targeting the V3 region of the 16S rRNA gene: 341F (5′-CCTACGGGAGGCAGCAG-3′) and 518R (5′GTATTACCGCGGCTGCTGG-3′) (0.25 µM each) (8). The 25 µl reaction for each sample was based on the Q5 high-fidelity DNA polymerase kit instruction provided by the

manufacturer (NEB Labs INC, Canada) with the exception that 1-2 µl of the 1:2, 1:5 and 1:10 diluted DNA was used as the template. The touchdown thermocycling program used was: 94ºC for 3 min, followed by 10 cycles of 94ºC for 30 s, 65-55ºC for 40 s, 72ºC for 1 min, followed by 20 cycles of 94ºC for 30 s, 55ºC for 40 s, 72ºC for 1 min; 53ºC for 40 s with a final elongation step at 72ºC for 10 min and a holding temperature at 4ºC. This PCR protocol was used for 16S rRNA gene amplification throughout this manuscript unless otherwise noted.

REFERENCES FOR THE SUPPORTING INFORMATION

1. Llamas, B., Valverde, G., Fehren-Schmitz, L., Weyrich, L. S., Cooper, A., Haak, W. From the field to the laboratory: Controlling DNA contamination in human ancient DNA research in the high-throughput sequencing era. STAR: *Science & Technology of Archaeological Research* **3**, 1-14 (2017).
2. Cooper, A., Poinar, H. N. Ancient DNA: Do It Right or Not at All. *Science* **289**, 1139-1139 (2000).
3. Porter, T. M., Golding, G. B., King, C., Froese, D., Zazula, G., Poinar, H. N. Amplicon pyrosequencing late Pleistocene permafrost: the removal of putative contaminant sequences and small-scale reproducibility. *Molecular Ecology Resources* **13**, 798-810 (2013).
4. D'Costa, V. M., King, C. E., Kalan, L., Morar, M., Sung, W. W. L., Schwarz, C., Froese, D., Zazula, G., Calmels, F., Debruyne, R., Golding, G. B., Poinar, H. N., Wright, G. D. Antibiotic resistance is ancient. Nature 477:457.
5. Bittelli M. 2011. Measuring soil water content: A review. *HortTechnology* **21**, 293-300 (2011).
6. Lim, C., Jackson, M. In methods of soil analysis. Part 2. Chemical and microbiological properties, 2nd ed SSSA, Madison, **WI**, 1-12 (1982).
7. Burt, R. Kellogg soil survey laboratory methods manual. United States Department of Agriculture, Natural Resources Conservation.
8. Monteux, S., Weedon, J. T., Blume-Werry, G., Gavazov, K., Jassey, V. E. J., Johansson, M., Keuper, F., Olid, C., Dorrepaal, E. Long-term in situ permafrost thaw effects on bacterial communities and potential aerobic respiration. *ISME* **12**, 2129-2141 (2018).
9. Vishnivetskaya, T. A., Petrova, M. A., Urbance, J., Ponder, M., Moyer, C.L., Gilichinsky, D. A., Tiedje, J. M. Bacterial Community in Ancient Siberian Permafrost as Characterized by Culture and Culture-Independent Methods. *Astrobiology* **6**, 400-414 (2006).
10. Bai, Y., Yang, D., Wang, J., Xu, S., Wang, X., An, L. Phylogenetic diversity of culturable bacteria from alpine permafrost in the Tianshan Mountains, northwestern China. *Research in microbiology* **157**, 741-751 (2006).
11. Rivkina, E., Shcherbakova, V., Laurinavichius, K., Petrovskaya, L., Krivushin, K., Kraev, G., Pecheritsina, S., Gilichinsky, D. Biogeochemistry of methane and methanogenic archaea in permafrost. *FEMS Microbiology Ecology* **61**, 1-15 (2007).
12. Johnson, S. S., Hebsgaard, M. B., Christensen, T. R., Mastepanov, M., Nielsen, R., Munch, K., Brand, T., Gilbert, M. T. P., Zuber, M. T., Bunce, M., Rønn, R., Gilichinsky, D., Froese, D., Willerslev, E. Ancient bacteria show evidence of DNA repair. *Proceedings of the National Academy of Sciences* **104**, 14401-14405 (2007).
13. Waldrop, M. P., Wickland, K. P., White, Iii, R., Berhe, A. A., Harden, J. W., Romanovsky, V. E. Molecular investigations into a globally important carbon pool: Permafrost-protected carbon in Alaskan soils. *Global change biology* **16**, 2543-2554 (2010).

14. Mackelprang, R., Waldrop, M. P., DeAngelis, K. M., David, M. M., Chavarria, K. L., Blazewicz, S. J., Rubin, E. M., Jansson, J. K. Metagenomic analysis of a permafrost microbial community reveals a rapid response to thaw. *Nature* **480**, 368 (2011).
15. Bischoff, J., Mangelsdorf, K., Gattinger, A., Schloter, M., Kurchatova, A. N., Herzschuh, U., Wagner, D. Response of methanogenic archaea to Late Pleistocene and Holocene climate changes in the Siberian Arctic. *Global Biogeochemical Cycles* **27**, 305-317 (2013).
16. Frank-Fahle, B. A., Yergeau, E., Greer, C. W., Lantuit, H., Wagner, D. Microbial functional potential and community composition in permafrost-affected soils of the NW Canadian Arctic. *PLoS One* **9**, e84761 (2014).
17. Hultman, J., Waldrop, M. P., Mackelprang, R., David, M. M., McFarland, J., Blazewicz, S. J., Harden, J., Turetsky, M. R., McGuire, A. D., Shah, M. B., VerBerkmoes, N. C., Lee, L. H., Mavrommatis, K., Jansson, J. K. Multi-omics of permafrost, active layer and thermokarst bog soil microbiomes. *Nature* **521**, 208 (2015).
18. Rivkina E, Petrovskaya L, Vishnivetskaya T, Krivushin K, Shmakova L, Tutukina M, Meyers A, Kondrashov F. 2016. Metagenomic analyses of the late Pleistocene permafrost – additional tools for reconstruction of environmental conditions. Journal Name: Biogeosciences; Journal Volume: 13; Journal Issue: 7; Conference: null; Patent File Date: null; Patent Priority Date: null; Other Information: null; Related Information: null:Medium: X; Size: 2207 to 2219; Quantity: null; OS: null; Compatibility: null; Other: null.
19. Bottos EM, Kennedy DW, Romero EB, Fansler SJ, Brown JM, Bramer LM, Chu RK, Tfaily MM, Jansson JK, Stegen JC. 2018. Dispersal limitation and thermodynamic constraints govern spatial structure of permafrost microbial communities. FEMS Microbiology Ecology 94.
20. Rivkina E, Friedmann E, McKay C, Gilichinsky D. 2000. Metabolic activity of permafrost bacteria below the freezing point. Appl Environ Microbiol 66:3230-3233.
21. Yergeau E, Hogues H, Whyte LG, Greer CW. 2010. The functional potential of high Arctic permafrost revealed by metagenomic sequencing, qPCR and microarray analyses. The Isme Journal 4:1206.
22. Wright GD, Poinar H. 2012. Antibiotic resistance is ancient: implications for drug discovery. Trends in Microbiology 20:157-159.
23. Tuorto SJ, Darias P, McGuinness LR, Panikov N, Zhang T, Häggblom MM, Kerkhof LJ. 2014. Bacterial genome replication at subzero temperatures in permafrost. The ISME journal 8:139.
24. Lydolph MC, Jacobsen J, Arctander P, Gilbert MTP, Gilichinsky DA, Hansen AJ, Willerslev E, Lange L. 2005. Beringian paleoecology inferred from permafrost-preserved fungal DNA. Appl Environ Microbiol 71:1012-1017.
25. Coolen MJ, van de Giessen J, Zhu EY, Wuchter C. 2011. Bioavailability of soil organic matter and microbial community dynamics upon permafrost thaw. Environmental microbiology 13:2299-2314.
26. Krivushin K, Kondrashov F, Shmakova L, Tutukina M, Petrovskaya L, Rivkina E. 2015. Two Metagenomes from Late Pleistocene Northeast Siberian Permafrost. Genome Announcements 3:e01380-14.

27. Coolen MJ, Orsi WD. 2015. The transcriptional response of microbial communities in thawing Alaskan permafrost soils. Frontiers in microbiology 6:197.
28. Mackelprang R, Burkert A, Haw M, Mahendrarajah T, Conaway CH, Douglas TA, Waldrop MP. 2017. Microbial survival strategies in ancient permafrost: insights from metagenomics. The Isme Journal 11:2305.
29. Gilichinsky D, Rivkina E, Bakermans C, Shcherbakova V, Petrovskaya L, Ozerskaya S, Ivanushkina N, Kochkina G, Laurinavichuis K, Pecheritsina S. 2005. Biodiversity of cryopegs in permafrost. FEMS microbiology ecology 53:117-128.
30. Hansen AA, Herbert RA, Mikkelsen K, Jensen LL, Kristoffersen T, Tiedje JM, Lomstein BA, Finster KW. 2007. Viability, diversity and composition of the bacterial community in a high Arctic permafrost soil from Spitsbergen, Northern Norway. Environmental microbiology 9:2870-2884.
31. Liebner S, Rublack K, Stuehrmann T, Wagner D. 2009. Diversity of aerobic methanotrophic bacteria in a permafrost active layer soil of the Lena Delta, Siberia. Microbial ecology 57:25-35.
32. Bellemain E, Davey ML, Kauserud H, Epp LS, Boessenkool S, Coissac E, Geml J, Edwards M, Willerslev E, Gussarova G. 2013. Fungal palaeodiversity revealed using high-throughput metabarcoding of ancient DNA from arctic permafrost. Environmental microbiology 15:1176-1189.
33. Penton CR, StLouis D, Cole JR, Luo Y, Wu L, Schuur EG, Zhou J, Tiedje JM. 2013. Fungal diversity in permafrost and tallgrass prairie soils under experimental warming conditions. Appl Environ Microbiol 79:7063-7072.
34. Treat CC, Wollheim WM, Varner RK, Grandy AS, Talbot J, Frolking S. 2014. Temperature and peat type control CO 2 and CH 4 production in Alaskan permafrost peats. Global change biology 20:2674-2686.
35. Mondav R, Woodcroft BJ, Kim E-H, McCalley CK, Hodgkins SB, Crill PM, Chanton J, Hurst GB, VerBerkmoes NC, Saleska SR. 2014. Discovery of a novel methanogen prevalent in thawing permafrost. Nature communications 5:3212.
36. Deng J, Gu Y, Zhang J, Xue K, Qin Y, Yuan M, Yin H, He Z, Wu L, Schuur EA. 2015. Shifts of tundra bacterial and archaeal communities along a permafrost thaw gradient in A laska. Molecular ecology 24:222-234.
37. Schostag M, Stibal M, Jacobsen CS, Bælum J, Taş N, Elberling B, Jansson JK, Semenchuk P, Priemé A. 2015. Distinct summer and winter bacterial communities in the active layer of Svalbard permafrost revealed by DNA-and RNA-based analyses. Frontiers in microbiology 6:399.
38. Wei S, Cui H, Zhu Y, Lu Z, Pang S, Zhang S, Dong H, Su X. 2018. Shifts of methanogenic communities in response to permafrost thaw results in rising methane emissions and soil property changes. Extremophiles 22:447-459.
39. Knoblauch C, Beer C, Liebner S, Grigoriev MN, Pfeiffer E-M. 2018. Methane production as key to the greenhouse gas budget of thawing permafrost. Nature Climate Change 8:309-312.
